# Supplementary material for: What cancer research makes the news? A quantitative analysis of online news stories that mention cancer studies
Source: PLoS One. 2021 Mar 10;16(3):e0247553. doi: 10.1371/journal.pone.0247553 (PMC7946182; doi:10.1371/journal.pone.0247553)
Supplement: Attachment — Submitted filename: PLOS ONE Response to Reviewers PONE-D-20-15200.pdf [file pone.0247553.s002.pdf]

Dear editors,

Again, we thank you and the reviewers for your feedback and suggestions regarding our manuscript entitled, "What cancer research makes the news? A quantitative analysis of online news stories that mention cancer studies" (Manuscript PONE-D-20-15200) by Moorhead, Krakow and Maggio. We are pleased to resubmit our manuscript with revisions based on this feedback. In the table below, we provide a description of how we addressed each reviewer comment. These revisions are also noted within the document using track changes.

**Editor comments**	**Author response**	**Edit location**
1. Add a data availability statement to the manuscript (see https://journals.plos.org/plosone/s/data-availability); make sure that the original data are accessible	We have reviewed the journal's data availability policy to ensure this submission complies with the minimal data set criteria and added the following data availability statement to the manuscript:<br><br>"Data for this study and related projects are freely available from this project's data repository. Please use the following link to access the data: https://zenodo.org/record/4075712 (see spss subfolder for project data and syntax files)."	p. 18
2. Please make sure that all the necessary supplementary information is provided and correctly referenced in the manuscript (please carefully read https://journals.plos.org/plosone/s/submission-guidelines and https://journals.plos.org/plosone/s/supporting-information).	Thank you for this comment. Because the data and syntax files for this project are located within our larger data portfolio on Zenodo, we have added more specific information in parentheses to direct readers to the relevant files for this manuscript and additionally clarified this information in an accompanying data availability statement per the journal's requirements.. We hope this clears up any confusion with locating the project files.	p. 18
3. The authors work with very low numbers, and therefore I believe that the data could be further	We revised the paragraph explainin how Altmetric.com retrieves mentions.	See additions made to p. 5 for an

San Francisco State University | Department of Journalism | 1600 Holloway Ave, SF, CA 94132

explored in order to describe, 1) how is Altmetric.com retrieving the mentions?, and 2) are these findings a reflection of what is happening (rare mentions to US-funded cancer research, or a limitation of the tool the authors are using?<br><br>For the first issue, simply going into the actual news mentioning the papers and looking at them (at least a sample, but the size of the data is so small, that it is manageable) to then **report how the news are linking to research and the context in which it is made would be enough**.<br><br>For the second, a potential idea would be to search in a news media database (the authors already mention two) for one of the more common cancers in a specific outlet (e.g., NY Times or any other), see how much they retrieve and see how much deals with research to find how they report research findings. If you find consistent evidence on them reporting in the way Altmetric.com identifies scientific literature, then you have some evidence on the robustness of	Also, we appreciate the reviewer's suggestion to better illustrate the content retrieved by Altmetric for readers. To this end, we conducted an exploratory analysis of all news mentions (n = 236) for the journal articles included in Table 4 (the top 10 journal articles by number of news mentions). We now include the following description of this content on page 8:<br><br>"We conducted an exploratory analysis of a subset of news mentions (n = 236 news stories from all journal articles included in Table 4) to illustrate the content included in Altmetric's categorization of the story as a "mention." Briefly, these news stories included both stories focused on summaries of the journal article as well as mentions of the journal article as part of a news story on a broader topic. Of these news stories, 59.5% represented original content written by a journalist at the news outlet, while 40.5% of news stories originated from a wire service such as the Associated Press. Eighty-two percent of news stories mentioned the name of the journal, such as JAMA or BMJ, and 74.9% mentioned the name of at least one co-author of the journal article. Most (69.0%) stories included a hyperlink to the specific journal article or its abstract on the journal's website."<br><br>In response to the reviewer's second suggestion, we also conducted a search of the terms "cancer research" and "lung cancer" across the study time period in the New York Times. This search returned 73 results for "cancer research" and 148 results for "lung cancer." Although this results list contained more news stories than those captured by Altmetric, an anecdotal examination of the returned search results revealed that a number of stories, such as book reviews, obituaries, and business/economics analyses, in this list but did not contain references to original peer-reviewed scientific research. Of the 4 news stories on lung cancer in the New York Times captured by Altmetric, all were also	improved explanation on how Altmetric.com retrieves mentions.<br><br>See p. 7 for the paragraph explaining the exploratory analysis.

your findings, even if it is anecdotal.	included in the search results on the New York Times website. Additionally, of the 50 stories on cancer research more broadly, we observed substantial overlap between Altmetric's list of mentions and stories retrieved by the New York Times' own search results for the same time period. These anecdotal findings reiterate our confidence that the news stories categorized as mentions by Altmetric's search criteria do align well with coverage on the news sites included in this study. While a more comprehensive comparative analysis is beyond the scope of this individual study, we appreciate the reviewer's suggestions and will consider how to apply this approach to future work focused on continued demonstration of the utility of Altmetric as a resource for scientific news coverage analysis.	
4. I'm still not convinced the chi-squared is worth keeping; consider removing. It adds very little, if anything, to the work and without examining standardized residuals it's hard to know what a significant chi-squared even means in this context. If the authors decide to keep it, please make sure the numbers in the sentences are clear and correct (what are the 204 and 240?) and the assumptions for chisquared are met.	We have revised the reporting of the chi squared analysis based on reviewer feedback and corrected the reported n in parentheses, which is now consistent with the output from the included data syntax files. We apologize for the earlier error in this reporting. We have also confirmed that assumptions for chi-squared tests have been met (e.g., no cells with less than 5 counts) and examined standardized residuals, which indicated the direction of the observed difference (greater coverage of common cancer types among traditional news sources compared to digital native sources). At the editor's discretion, we would be happy to remove this brief portion of the analysis and instead simply report this as a descriptive finding without the chi squared test, e.g. "Total mentions of at least one common cancer type differed across traditional and digital native news sources. Traditional news sources included more mentions of research on common cancer types (n = 127) compared to digital native mentions (n = 71)."	See p. 7.
5. Figure 1, the ratios are a good idea, although the Figure then loses the impact about how serious each	We thank the reviewer for providing thoughtful feedback regarding how to enhance the clarity of Figure 1. Following the provided feedback, we have flipped the figure's coordinates for increased	See p. 9.

cancer is in terms of causing death. I'm including a version of the graph I sent in R in the previous review in Excel with the following adjustments from what was included in the paper: (1) flip the coordinates so that the labels are easier to read (2) order the bars by height (3) add some way of knowin g how serious each cancer is (either a second bar showing deaths/each cancer or some kind of labeling)	readability of labels and reordered the bars by height. In combination with Table 2, we believe these presentations of the data now give a clearer picture of the severity of each cancer type as well as the degree of news mentions per estimated cancer death.	
6. I can't figure out where the code is for this paper from the zenodo link that was sent; what is saved there appears to be mostly stuff from the prior paper?	We have added brief wording to point readers toward the specific portion of our data repository containing the project files for this specific manuscript while also providing access to the data files associated with our larger research initiative: "(see SPSS subfolder for data and syntax files)."	See pgs. 6 and 18.

We greatly appreciate your further review of our manuscript and hope that the revisions as described above and in the revised paper meet your expectations. Please do not hesitate to contact me with any questions or additional requests.

Best regards,

Laura Moorhead

Dr. Laura Moorhead
Assistant Professor, Journalism
